# Distinct antibody responses of patients with mild and severe leptospirosis determined by whole proteome microarray analysis

**DOI:** 10.1371/journal.pntd.0005349

**Published:** 2017-01-31

**Authors:** Carolina Lessa-Aquino, Janet C. Lindow, Arlo Randall, Elsio Wunder, Jozelyn Pablo, Rie Nakajima, Algis Jasinskas, Jaqueline S. Cruz, Alcineia O. Damião, Nívison Nery, Guilherme S. Ribeiro, Federico Costa, José E. Hagan, Mitermayer Galvão Reis, Albert I. Ko, Marco Alberto Medeiros, Philip L. Felgner

**Affiliations:** 1 Fiocruz, Bio-Manguinhos, Brazilian Ministry of Health, Rio de Janeiro, RJ, Brazil; 2 Fiocruz, Gonçalo Moniz Research Institute, Brazilian Ministry of Health, Salvador, BA, Brazil; 3 Department of Epidemiology of Microbial Diseases, Yale School of Public Health, New Haven, CT, United States of America; 4 Antigen Discovery Inc, Irvine, CA, United States of America; 5 Department of Medicine, Division of Infectious Disease, University of California Irvine, Irvine, California, United States of America; 6 Institute of Collective Health, Federal University of Bahia, Salvador, BA, Brazil; University of California San Diego School of Medicine, UNITED STATES

## Abstract

**Background:**

Leptospirosis is an important zoonotic disease worldwide. Humans usually present a mild non-specific febrile illness, but a proportion of them develop more severe outcomes, such as multi-organ failure, lung hemorrhage and death. Such complications are thought to depend on several factors, including the host immunity. Protective immunity is associated with humoral immune response, but little is known about the immune response mounted during naturally-acquired *Leptospira* infection.

**Methods and principal findings:**

Here, we used protein microarray chip to profile the antibody responses of patients with severe and mild leptospirosis against the complete *Leptospira interrogans* serovar Copenhageni predicted ORFeome. We discovered a limited number of immunodominant antigens, with 36 antigens specific to patients, of which 11 were potential serodiagnostic antigens, identified at acute phase, and 33 were potential subunit vaccine targets, detected after recovery. Moreover, we found distinct antibody profiles in patients with different clinical outcomes: in the severe group, overall IgM responses do not change and IgG responses increase over time, while both IgM and IgG responses remain stable in the mild patient group. Analyses of individual patients’ responses showed that >74% of patients in the severe group had significant IgG increases over time compared to 29% of patients in the mild group. Additionally, 90% of IgM responses did not change over time in the mild group, compared to ~51% in the severe group.

**Conclusions:**

In the present study, we detected antibody profiles associated with disease severity and speculate that patients with mild disease were protected from severe outcomes due to pre-existing antibodies, while patients with severe leptospirosis demonstrated an antibody profile typical of first exposure. Our findings represent a significant advance in the understanding of the humoral immune response to *Leptospira* infection, and we have identified new targets for the development of subunit vaccines and diagnostic tests.

## Author summary

Leptospirosis is zoonotic disease of global importance, with over a million cases and nearly 60,000 deaths annually. Symptomatic disease presentation ranges from a mild febrile disease with non-specific symptoms to severe forms, characterized by multi-organ failure, lung hemorrhage, and death. Factors driving severe outcomes remain unclear, but the host immune response likely plays an important role. In the present study, we applied high throughput techniques to identify the antibody profiles of patients with severe and mild leptospirosis. We discovered a limited number of immunodominant antigens, specific to patients. Surprisingly, we found the antibody repertoire varies in patients with different clinical outcomes and hypothesized that patients with mild symptoms were protected from severe disease due to pre-existing antibodies, while the profile of patients with severe outcomes was representative of a first exposure. These findings represent a substantial step forward in the knowledge of the humoral immune response to *Leptospira* infection, and we have identified new targets for vaccine and diagnostic test development.

## Introduction

Leptospirosis causes over one million cases and nearly 60,000 deaths annually, with the greatest disease burden in urban slums in tropical and subtropical countries [1–3]. Ten pathogenic *Leptospira* species, over 200 serovars, and a large number of mammalian reservoirs, including rats, have facilitated the emergence of leptospirosis as a major, global public health problem. Humans typically become infected through direct contact with reservoir urine-contaminated soil or water, and develop a broad spectrum of clinical manifestations, including hepato-renal failure and pulmonary hemorrhage syndrome in severe cases, which have high mortality rates [2, 4–6]. The factors contributing to disease severity remain poorly understood, but bacterial virulence, inoculum dose and the host immune response are thought to play important roles in development of severe outcomes [2, 4].

Experimental animal models of *Leptospira* infection have provided a majority of evidence that antibodies play a key role in protection against and clearance of *Leptospira* infection [7–9]. Passive transfer of whole cell leptospiral vaccine and specific anti-leptospiral antibodies (Ligs) are protective against homologous infection in animal models, demonstrating antibodies are sufficient for immunity against experimental homologous infection [10–13]. Additionally, antibodies against LPS are serovar-specific, are correlated with agglutinating antibody titers, and confer limited cross-protection against other serovars [14, 15]. Several studies have shown that leptospirosis patients develop a robust antibody response during infection, especially anti-LPS antibodies, which correspond to the majority of the antibodies produced [12, 16, 17].

The large number of pathogenic *Leptospira* serovars and poor cross-protection observed for anti-LPS antibodies, have made the identification of anti-*Leptospira* protein antibodies a high priority for vaccine and diagnostic test development [18, 19]. In support of this, immunization with an LPS-deficient *Leptospira* strain in experimental animal models conferred cross-protection, implicating anti-protein and other immune responses in protection against infection. [19] Additionally, our group has applied a protein microarray methodology to evaluate the antibody repertoire generated in natural *Leptospira* infection and identified strong antibody responses in healthy exposed individuals as well as several IgG serodiagnostic antigens specific to patients [20, 21].

Analyses of antibody immune responses against infectious agents are essential not only for diagnostic and vaccine development, but also to providing insight in the mechanisms involved in pathogenicity [22]. Protein arrays are an excellent platform that allow for the screening of antibody protein targets in a high-throughput manner, with high sensitivity and high specificity [22–24]. These elements facilitate the assessment of many analytes simultaneously and allow for the identification, quantification and comparison of individual antigenic responses following exposure to microorganisms. Our group has efficiently employed high-density proteome arrays in the characterization of antibody signatures against several infectious agents of human and veterinary importance [25–30], including *Leptospira interrogans* and other spirochetes [21, 31].

In the current study, we used a whole genome proteome microarray approach to describe the first comprehensive profile of the human antibody response to symptomatic *Leptospira* infection. We probed 192 serum samples including patients with different clinical outcomes and healthy controls, and compared their antibody profiles against *L*. *interrogans* serovar Copenhageni proteins, the serovar associated with >90% of the urban leptospirosis cases in Salvador, Brazil [32, 33]. We identified promising candidates for the development of new diagnostic tests and subunit vaccines and discovered different antibody profiles, which associated with disease severity. Lastly, the antibody kinetics suggest a majority of patients with severe leptospirosis likely have a primary infection, while those with milder disease have evidence of a secondary infection. Our results provide novel insights into the complexity of the immunity in naturally-acquired leptospirosis as well as new diagnostic test candidates.

## Methods

### Ethics statement

The study protocol was approved by the institutional review board committees of Yale University and Oswaldo Cruz Foundation prior to study initiation. All participants provided written informed consent in their native language prior to sample and data collection. All samples were anonymized before research use.

### Study enrollment and sample collection

All 61 patient samples were collected during active surveillance for acute leptospirosis at the Hospital Couto Maia (31 severe group patients) and the São Marcos Emergency Clinic (30 mild group outpatients) in Salvador, Brazil between years 2005–2011. Laboratory confirmation was defined as positive microagglutination test (seroconversion, four-four rise in titer, or single titer ≥ 1:800) and/or positive ELISA and/or positive PCR for *Leptospira* DNA as previously described [32]. Serum samples from patients with mild or severe leptospirosis were collected twice: (i) acute sample, collected at patient admittance at the health care unit and (ii) convalescent sample, collected 5–276 days after the first sampling. Controls consisted of (i) 37 sera from healthy *Leptospira*-unexposed (naïve) volunteers from California/US and (ii) 37 sera from healthy participants enrolled in a cohort study in a high risk urban slum community in Salvador, endemic for leptospirosis.

### *Leptospira* ORF amplification and high throughput cloning

The entire ORFeome of *Leptospira interrogans* serovar Copenhageni strain Fiocruz L1-130 was amplified by PCR and cloned into pXI vector using a high-throughput PCR recombination cloning method developed by our group [34]. In this strategy, cloned ORFs were expressed with C-terminal hemaglutinin (HA) tag and N-terminal poly-histidine (His) tag. Genes larger than 3kb were cloned as smaller segments as described previously [20, 21] and the *ligA* and *ligB* genes (LIC10465 and LIC10464, respectively) were fragmented according to the repeated Big domains present in the structure of each protein (LigB Repeats 7–12, LigA Repeats 7–13 and LigA/B Repeats 1–6) [35]. After identifying the seroreactive antigens on the microarrays, the inserts in the corresponding plasmids were confirmed by nucleotide sequencing by the Sanger method.

### Microarray probing

Microarray fabrication was performed as described previously [20, 21]. Briefly, purified mini-preparations of DNA were used for expression in *E*. *coli in vitro* based transcription-translation (IVTT) reaction system (RTS Kit, Roche), following the manufacturer´s instructions. Negative control reactions were those performed in the absence of DNA template (“NoDNA” controls). Protease inhibitor mixture (Complete, Roche) and Tween-20 (0.5% v/v final concentration) were added to the reactions, which were then printed onto nitrocellulose coated glass FAST slides using an Omni Grid 100 microarray printer (Genomic Solutions). Multiple negative control reactions and positive control spots of an IgG mix containing mouse, rat and human IgG and IgM (Jackson Immuno Research) were added to the arrays. Protein expression was verified by probing the array with monoclonal anti-polyhistidine (Sigma Aldrich) and anti-hemaglutinin (Roche Applied Science) as previously described [20, 21].

Human sera samples were diluted 1/100 in Protein Array Blocking Buffer (Whatman) supplemented with 10% v/v *E*. *coli* lysate 10mg/mL (McLab) and incubated 30 min at room temperature (RT) with constant mixing prior to addition to the microarray. Arrays were blocked for 30 min with Protein Array Blocking Buffer and then incubated with diluted samples overnight at 4°C, with gentle rocking. Washes and incubation with conjugate antibodies were performed as described previously [20, 21]. Slides were scanned in a Perkin Elmer ScanArray confocal laser and intensities were quantified using QuantArray package.

### Cloning, expression and purification of recombinant proteins

Selected ORFs were cloned into pET100-TOPO plasmid (Invitrogen) for His-tagged recombinant protein expression in BL21 (DE3) Star *E*. *coli* cells, according to the manufacturer’s recommendations. Recombinant protein expression was performed with EnPresso B system (Biosilta). Briefly, pre-cultured cells were inoculated 1/100 into 3.5 mL of EnPresso B medium supplemented with Ampicilin 100 μg/mL Reagent A 1.5 U/μL and grown shaking (160 rpm) at 30°C for 16–18 hs in 24-well culture blocks. Expression was induced by the addition of 350 μL of the booster reagent supplemented with 15U/μL Reagent A and 100 mM IPTG, for 24 h at 30°C under 160 rpm shaking. Cells were then harvested and lysed with 0.05 g of Cellytic Express (Sigma) for each mL of final culture, for 30 min at RT. Lysates were applied to a Ni^2+^-charged resin (Qiagen) and recombinant proteins were manually purified using 20mM Tris (pH 8.0) buffers with increasing concentrations of Imidazole. Washes varied from 5 mM to 40 mM Imidazole, depending on the protein, and elution was performed with 500 mM or 1M Imidazole. Imidazole was removed by dialysis (Thermo Scientific dialysis cassettes) and the purified proteins were checked for homogeneity in 12.5% SDS-PAGE. Protein concentration was determined by the BCA method (Thermo Scientific) according to the manufacturer's recommendations.

### Multi-antigen print immunoassay

The assay was performed as described previously [23]. Briefly, 100 ng of each purified protein was immobilized on a nitrocellulose membrane strip. A semi-automatic micro-aerolization device was used to generate parallel bands with no visible marks. The membrane was cut into 0.5 cm wide strips perpendicularly to the antigen bands. The strips were blocked for 90 min with 4% reduced-fat bovine milk diluted in PBST (PBS + 0.5% Tween 20) and then incubated for 1 h at RT with individual serum samples diluted 1:200 in PBST 0.25% BSA and 5% v/v *E*. *coli* lysate 20mg/mL. After 3 washes with PBST, the strips were incubated for 1 hour with alkaline phosphatase–labeled anti-human IgG antibody (Sigma-Aldrich) diluted 1:30.000 in PBST 0.25% BSA. The strips were then washed 3 times with PBST and revealed with Western Blue Stabilized Substrate for Alkaline Phosphatase (Bio-Rad) for 10 min. The reaction was stopped with distilled water. Strips were air-dried and scanned images were converted to gray scale before band intensity quantification with ImageJ software (found at http://rsbweb.nih.gov/ij/).

### Protein array data analysis

Array signal intensity was quantified using QuantArray software. Spots intensity raw data were obtained as the mean pixel signal intensity with automatic correction for spot-specific background. Data was normalized by dividing the raw signal for each IVTT protein spot by the median of the sample-specific IVTT control spots (fold-over control [FOC]) and then taking the base-2 logarithm of the ratio (log2 FOC). Conceptually, a normalized signal of 0.0 is equal to control spot signal, and a normalized signal of 1.0 is 2-fold higher than control spot signal.

When evaluating a protein spot as reactive or non-reactive, normalized signals >1.0 were considered reactive. These designations were used to evaluate response frequency and to identify a subset of sero-reactive proteins for further analysis. A given protein on the array was considered sero-reactive if it was reactive in at least 60% of the samples in one or more of the following groups: severe disease, acute sample (n = 30); severe disease, convalescent sample (n = 30); mild disease, acute sample (n = 30); mild disease, convalescent sample (n = 30); endemic controls (n = 30); naïve controls (n = 30). Sero-reactive proteins were identified separately using IgG and IgM responses.

For each sero-reactive protein, sample groups were compared using t-tests [R stats package] and the area under receiver operator characteristic curve (AUC) [R rocr package]. Proteins with t-test p-value < 0.05 after correction for false discovery [36] and AUC > 0.70 were identified as differentially reactive.

### Clinical data analysis

Clinical features of the leptospirosis patients participating in this study were described using frequencies and medians with interquartile (IQR) ranges calculated in Excel (Table 1). The Fisher Exact test or the Mann-Whitney test were used to compare clinical presentations of patients with mild or severe disease using GraphPad Prism 5.02 software.

**Table 1:** Clinical characteristics for patients with mild or severe leptospirosis.

CHARACTERISTICS		MILD		SEVERE	
	N	Median (IQR) or N (%)	N	Median (IQR) or N (%)	p-value
**Demographics**					
Male sex	30	16.0 (53.3)	31	27.0 (87.0)	**0.003**
Age	29	26.5 (17.3–36.8)	30	31.0 (24.5–48.8)	**0.039**
**Clinical Presentation**^**a**^					
Days of symptoms^b^					
Acute phase	29	5.5 (3.0–7.8)	30	7.0 (6.0–9.0)	0.085
Convalescent phase	29	44 (22.0–69.5)	30	27.0 (22.5–61.5)	0.681
Hematocrit (%)	12	38.5 (34.5–44.5)	31	34.0 (29.0–37.0)	**0.027**
Platelet count (1000/μL)	30	217.0 (154.3–238.0)	31	73.0 (62.5–177.5)	**0.011**
**Laboratory Confirmation**					
Agglutinating Antibody Titers					
Acute phase	30	0 (0–175)	31	200 (0–2400)	**0.013**
Convalescent phase	30	300 (0–800)	31	3200 (1600–6400)	**0.0001**
**Outcomes**					
Respiratory failure^c^	30	0	31	2 (6.5)	0.492
ICU admission	30	0	31	7 (22.5)	**0.011**
Oliguric renal failure^d^	30	0	31	24 (77.4)	**<0.0001**

^a^Values at time of hospital or clinic admission.

^b^Prior to sample collection.

^c^Respiratory failure was defined as presence of pulmonary hemorrhage (>250 ml) or mechanical ventilation.

^d^Oliguric renal failure was defined as oliguria (<500mL urine/day) or anuria (<50ml urine/day) or patient received hemodialysis.

### Microarray data accession number

The raw and normalized array data used in this study have been deposited in the Gene Expression Omnibus archive (www.ncbi.nlm.nih.gov/geo/), accession number GSE86630.

## Results

### Patient clinical and laboratory characteristics

To identify antigens associated with symptomatic leptospirosis and severe disease (requiring hospitalization), we enrolled 31 patients hospitalized with suspected leptospirosis, 30 individuals treated at an urgent care facility for suspected leptospirosis, 30 individuals living in the same communities as enrolled patients (hyperendemic controls), and 30 unexposed controls (naïve controls). All patients survived and provided paired acute and convalescent sera samples. Table 1 describes patient characteristics for clinical and biochemical tests performed during hospitalization or outpatient treatment. Hospitalized patients presented with more severe disease: 77.4% had oliguric renal failure, 6.5% had respiratory failure, and 22.5% required ICU admission, while none of these outcomes were observed in outpatients. Additionally, the agglutinating antibody titers for hospitalized patients were significantly higher during acute illness and convalescence compared to patients with mild leptospirosis (*p* = 0.011; *p* = <0.0001). However, while hospitalized patients (severe disease) were older (*p* = 0.039) and predominantly male (*p* = 0.03), there were no significant differences in days of symptoms at acute or convalescent sample collections between patients with mild and severe leptospirosis (acute *p* = 0.085; convalescent *p* = 0.681). Therefore, any differences observed in outcomes were not due to duration of illness or sampling times.

### Identification of potential leptospirosis serodiagnostic antigens

**Fig 1:**
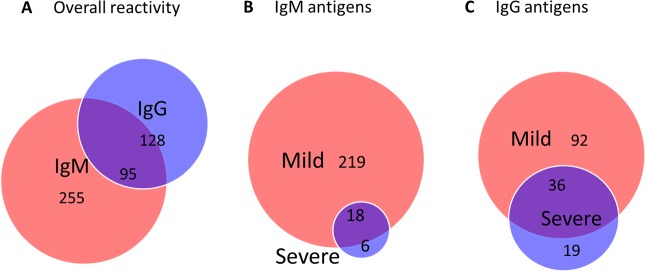
Overall IgM and IgG antibody recognition of leptospiral proteins. (A) Venn diagrams of IgM and IgG leptospiral proteins recognized by humans. Overlap of IgM (B) or IgG (C) sero-reactive antigens identified in patients with mild and severe leptospirosis.

In order to determine whether there is an antibody signature specific to symptomatic disease, we probed the protein arrays with a collection of 192 sera samples, including leptospirosis patients and healthy individuals living in areas with or without endemic transmission of leptospirosis. IgM and IgG probing revealed a set of 478 reactive antigens for both acute and convalescent phases, corresponding to 12.5% of all 3819 proteins and segments included on the arrays. Of these, 255 were specific for IgM, 128 were specific for IgG and 95 were recognized by both antibodies (Fig 1A). Interestingly, we detected a majority of the IgM and IgG antigens in patients with mild disease (Fig 1B and 1C). To identify antigens specific to patients with confirmed leptospirosis (serodiagnostic antigens), we then compared antigens from the sera of patients with those from healthy individuals and found 36 antigens with significantly higher IgG reactivity in leptospirosis patients than in healthy volunteers from United States or healthy individuals living in a highly endemic area in Brazil. Of these, 12 (33%) were identified during acute leptospirosis (S2 Table) and 33 (92%) during convalescence (S3 Table).

**Fig 2:**
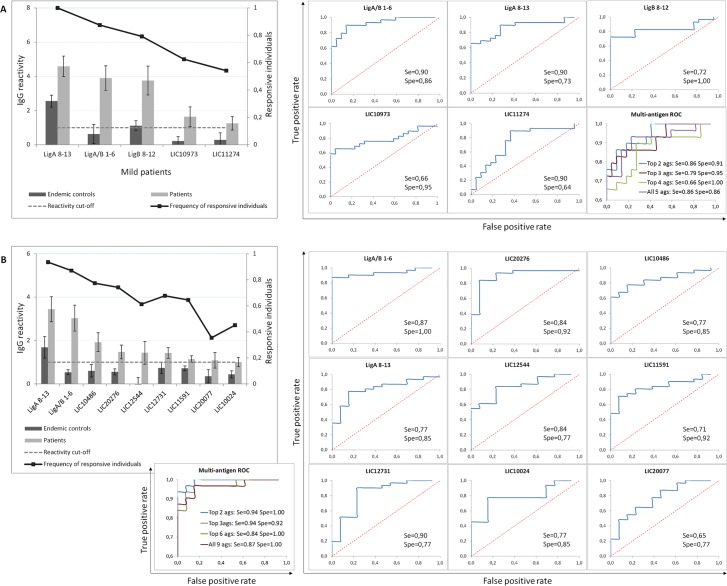
Serodiagnostic antigens identified for patients with mild and severe leptospirosis. Histograms plot the average normalized intensity (Y axis) of each antigen (X axis) for hyperendemic controls (dark gray bars) and patients with mild (A) or severe (B) disease (light gray bars), with the frequency of responsive individuals (black line, secondary axis). Error bars indicate S.E. Single or multi-antigens ROC curves of the identified serodiagnostic antigens for mild (A) or severe (B) groups are shown with sensitivity and specificity rates.

Early antigen detection during infection is critical for the development of a new diagnostic test for leptospirosis. Therefore, we first focused on serodiagnostic antigens identified during acute phase in patients with mild or severe disease. Surprisingly, we found only a limited subset of all the seroreactive antigens were significantly recognized by IgGs in patients relative to endemic and naïve control volunteers: 11 of the 128 in the mild patient group and 28 of the 55 in the severe group (Fig 2A). Of these only 5 of the 11 and 9 of the 28 were present during acute illnesss. For the mild group, the Lig proteins were the antigens with highest accuracy, especially LigA/B 1–6, with 90% sensitivity, 86% specificity and AUC of 0.916. To determine whether we could increase both sensitivity and specificity by combining the antigens, we constructed Receiver Operating Characteristic (ROC) curves for combinations of the 5 antigens to assess antigens diagnostic performance (Fig 2A). We found that combining the top two antigens LigA/B 1–6 and LigA 8–13 yielded slightly higher sensitivity (86%) and specificity (91%) than the other combinations (Fig 2A). We performed similar analyses for the 9 antigens specific to the severe group. Again, the best diagnostic accuracy was achieved with LigA/B 1–6 (AUC = 0.935, 87% sensitivity, 100% specificity) followed by LIC20276 (AUC = 0.901, 84% sensitivity, 92% specificity). When we combined both antigens, sensitivity reached 94%, and specificity was 100% (Fig 2B). For the remaining antigens, sensitivity ranged from 77% to 90% and specificity ranged from 77% to 92%. Again, other combinations did not yield better combined sensitivity and specificity (Fig 2B). Our results indicate that we have identified candidates for new leptospirosis diagnostic tests and have discovered that there may be a limited dominant antigen antibody response to *Leptospira* infection.

### Identification of potential subunit vaccine candidates in recovered leptospirosis patients

We analyzed the responses from convalescent sera to determine whether there were major shifts in antibody responses to specific antigens with time. Patients recovering from mild disease had significantly higher IgG titers for 10 antigens compared to endemic controls, while the number of antigens nearly tripled for patients with severe clinical presentation (S3 Table). Antigens identified at convalescent phase accounted for ~92% of all diagnostic antigens (33 in 36 total IgG antigens) and LigA/B 1–6 and LigB 8–12 were the antigens with best diagnostic performance for patients with severe and mild disease, respectively. While these antigens do not have diagnostic potential, they do represent possible subunit vaccine candidates as robust antibody responses were generated over the duration of illness.

### Protein microarray validation by MAPIA

**Fig 3:**
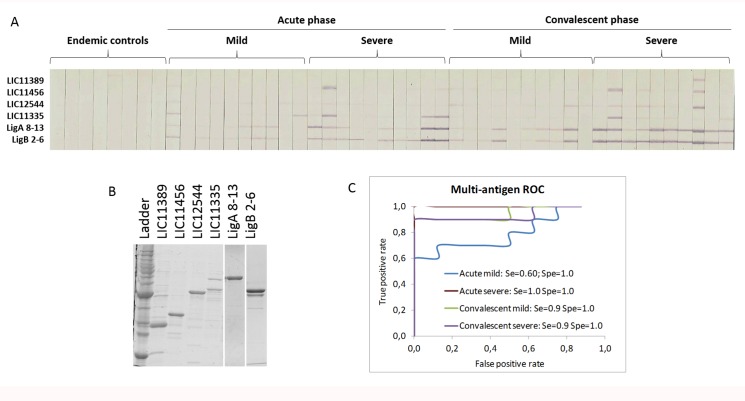
Validation of microarray results by MAPIA. (A) MAPIA strips probed for specific IgG in leptospirosis patients and endemic controls. Strips are grouped by disease severity, in acute and convalescent phases. (B) SDS PAGE of the 6 purified recombinant proteins that were applied to MAPIA strips. (C) ROC curves of the combination of all 6 antigens are shown for mild and severe groups at both acute and convalescent phases.

To confirm the diagnostic and subunit vaccine potential of the sero-reactive antigens detected on the microarray chips, we purified six proteins from *E*. *coli* BL21 *in vitro* (Fig 3B), and printed onto nitrocellulose membranes. We probed the immunostrips with serum from 8 endemic controls and 20 acute-phase patients, of which 10 had mild disease and 10 had severe disease. Serum from leptospirosis patients showed greater reactivity than serum from controls, especially serum from severe patients at convalescent phase (Fig 3A). To assess the ability of these six antigens to distinguish between patients and controls, a multi-antigen ROC curve was generated (Fig 3C), and demonstrated that the six selected antigens yielded a specificity of 100% and a sensitivity of 60% for acute mild group and 90% for the remaining groups.

### Distinct antibody profiles associated with disease presentation

As there is limited knowledge of the factors contributing to leptospirosis severe disease outcomes, we compared the antibody kinetics of patients to determine whether there are differences in antibody responses based on disease severity. We first compared the global IgG and IgM reactivities against all 478 reactive antigens identified in the microarrays by comparing the summed average signal intensities for each antigen during acute illness with that at convalescence. We detected a trending increase in IgG reactivity in patients with severe leptospirosis, which reached statistical significance when we analyzed the signals from the 36 patient-specific antigens (p<0.05) (S2 Fig). We did not observe this trend in patients with mild disease. For IgM-specific antigens, we observed no significant differences for either patient group or antigen set (S2 Fig). Thus, we identified significant IgG responses increases only in the severe patient group over time.

**Fig 4:**
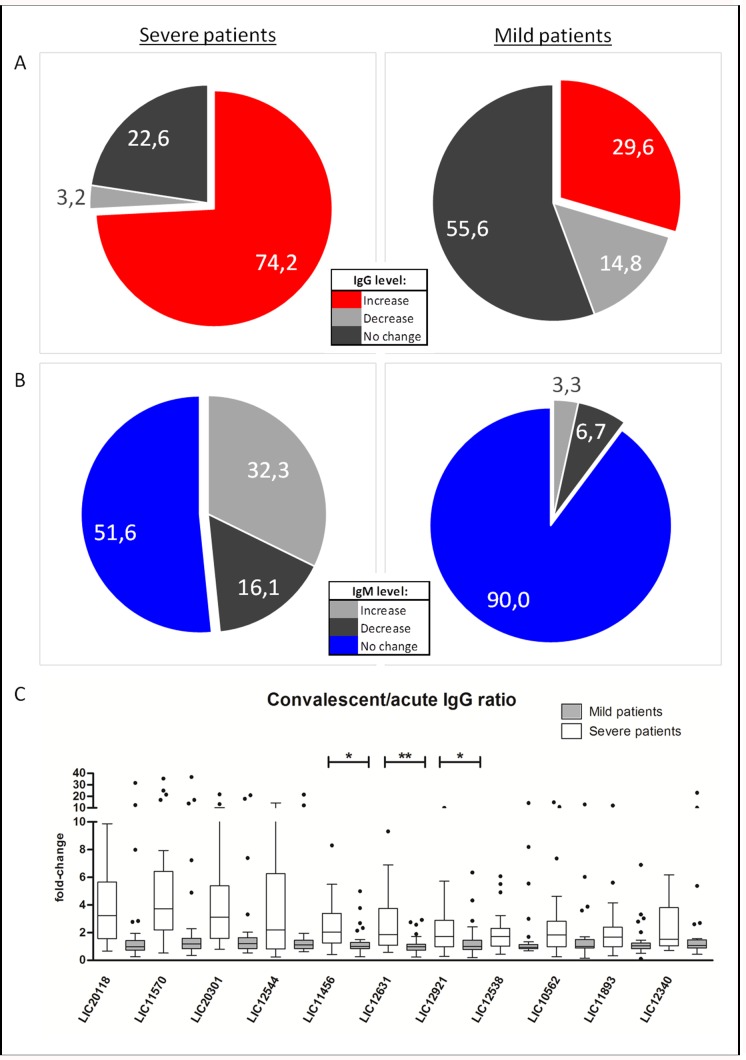
IgM and IgG antibody kinetics in patients with mild and severe leptospirosis. Percentage of patients that showed increase, decrease or unchanged IgG (A) and IgM (B) levels from acute to convalescent phases. Patients with severe disease are shown on the left and patients with mild form, on the right. (C) Boxplot shows the IgG fold-change (y-axis) of mild (dark gray) and severe (light gray) groups for each of the antigens in the x-axis. Significant differences are marked with star (*p<0.01; **p<0.001).

To understand the differences in antibody kinetics in patients in more detail, we next compared the antibody responses to the 36 differentially reactive antigens at the acute and convalescent time points for each individual by two way t-test. Based on the results of each t-test the individuals were categorized as: (i) increasing, when average response to the 36 differentially reactive antigens was higher at convalescent time point than acute, and p-value < 0.05), (ii) no change (p-value > 0.05) or (iii) decreasing, when average response to the 36 differentially reactive antigens was lower at convalescent time point than acute, and p-value < 0.05. This comparison yielded vastly different profiles for patients with mild disease and severe disease. When analyzing IgG responses, we categorized 74.4% of patients with in the severe group as “increasing” versus only 29.6% of patients in the mild group (Fig 4A). When analyzing IgM responses, we categorized 32.3% of patients in the severe group as “increasing” versus only 3.3% in the mild group (Fig 4B). Additionally, 90.0% of IgM responses did not change over time in the mild group, compared to 51.6% in the severe group. Altogether, these data clearly demonstrate that leptospirosis patients with different clinical presentations generate distinct antibody profiles.

In our kinetic antibody analyses, we enrolled five patients with mild leptospirosis, which had antibody profiles that resembled those of patients with severe leptospirosis: all had increases in IgG levels over time for 10 antigens (Fig 4C and S2). Though these five patients clearly developed an antibody response more representative of patients with severe disease (S3 Fig), including a higher convalescent agglutinating antibody titer (400–12800), they did not present with any severe clinical outcomes we measured. All other clinical and laboratory features were similar to the 25 patients with mild leptospirosis (S4 Table).

## Discussion

Leptospirosis is a disease with a broad spectrum of clinical manifestations ranging from asymptomatic and nonspecific acute febrile illnesses to life-threatening renal failure or pulmonary hemorrhage syndrome [2, 37]. Over a million cases of severe leptospirosis occur every year. This figure represents only a faction (potentially 5–15%) of the total mild leptospirosis cases, which usually are not identified by surveillance systems. The mechanisms involved in poor disease progression remain poorly defined, but pathogen related and host factors likely contribute to this heterogeneity [2, 4]. Here, we identified 12 specific IgG antigens that differentiate acute symptomatic disease from uninfected individuals in endemic regions and therefore represent promising diagnostic candidates for an early laboratory test for the diagnosis of leptospirosis. We also identified patient-specific antigens during convalescence, which are putative subunit vaccine candidates. Lastly, we showed that patients with different clinical presentations generate distinct antibody kinetic profiles, and we hypothesize that since antibodies are protective, disease severity and the antibody signatures may indicate primary and secondary infections.

We identified 12 IgG serodiganostic antigens for acute leptospirosis. Among them are the well-known sero-reactive proteins LigA/B 1–6, LigA 8–13, LigB 8–12 and LIC10973 (OmpL1). Several published studies used the Ligs as diagnostic markers for leptospirosis [35, 38–41] as well as OmpL1, especially in combination with LipL21, LipL32 or LipL41 [42]. Our group has previously identified LIC10486 (hypothetical protein) and LIC12544 (DNA binding protein) using the protein microarray platform [21]. The remaining 6 proteins LIC10024 (adenylate/guanylate cyclase), LIC11591 (exodeoxyribonuclease VII large subunit), LIC20077 (polysaccharide deacetylase) and the hypothetical proteins LIC11274, LIC20276 and LIC12731 are promising newly identified serodiagnostic antigens, especially LIC20276, which improved diagnostic performance for severe disease in combination with LigA/B 1–6. Interestingly, patients showed antibody reactivity against several proteins annotated as hypothetical proteins, not only at acute disease, but also during convalescence. These results indicate that even though these proteins have not been assigned any function, they are indeed expressed by the bacteria and might play an important role in host infection. Further studies should be done in order to evaluate these antigens performance in different diagnostic platforms, such as ELISA and rapid tests. For diagnostic purposes, a complete validation study needs to be performed, including the probing of a more extensive sample collection, comprising more leptospirosis patients as well as healthy controls and patients with other febrile illness, such as dengue, sifilis and hepatitis A.

The results presented here are consistent with our previous findings [21]. We detected 13 of the 24 IgG antigens previously found in hospitalized patients, strengthening the diagnostic potential of those antigens and validating the protein microarray antigen discovery platform. The inclusion of 39% of *L*. *interrogans* predicted ORFeome, however, did not provide significant advantage in diagnostic antigen discovery, since only 3 out of the 1489 proteins and segments added to the microarray were serodiagnostic, indicating that the algorithm used by our group to select the proteins included in the partial microarray was effective. Indeed, 32 out of the 36 diagnostic antigens identified here fall in at least one of the enrichment categories described by our group for antibody recognition [20, 43, 44].

Leptospirosis patients and healthy controls reacted against 12% of the *L*. *interrogans* predicted ORFeome. The majority of the imunodominant antigens were IgM specific, which corresponded to >50% of the sero-reactive proteins. The high number of IgM antigens may reflect the broad and low-affinity antigen-antibody interaction typical of IgM antibodies [45, 46]. These features usually make IgM a hard indicator of reliable diagnostic tests and might have hindered the identification of IgM diagnostic targets, as they usually account for lower specificity in IgM-based serological tests and high background reactivity in negative samples [45]. Here, we had great success in detecting IgG antigens with potential use as diagnostic or vaccine targets, but further studies are needed to identify IgM antigens.

In our previous work, we have shown that healthy individuals who live in areas with endemic transmission of leptospirosis have a background IgG reactivity against leptospiral protein antigens, possibly due to the constant exposure to the pathogen [21]. As it is well known that antibodies are one of the main immune mechanisms in naturally-acquired leptospirosis [16], the presence of high IgG levels in such individuals suggests that those antibodies might play an important role in protection against the development of clinical leptospirosis. Despite this background IgG reactivity, we were able to identify antigens for which IgG levels were even higher among hospitalized leptospirosis patients, especially at the patient's convalescent sample [21]. Indeed, most of the 36 serodiagnostic antigens identified in the present study were detected in the convalescent sample of patients with severe disease. A considerably smaller number of antigens was detected in patients with the mild form, suggesting that their IgG antibody response is more similar to healthy individuals living in the same area.

The distinct antibody profiles associated with each group were not due to differences in days of symptoms. We hypothesize that patients with mild leptospirosis had a background IgG reactivity that protected them from severe clinical manifestations while the lack of such IgG response might have favored the development of severe outcomes in hospitalized patients. In general, the first contact with an infectious agent is serologically characterized by a gradual increase in IgM, with a peak on days 7–10 after pathogen exposure, followed by an increase in IgG on days 10–14. In a secondary infection, however, a robust IgG response is rapidly mounted as a consequence of the activation of memory B cells generated during the primary infection [47–49]. In the light of this, the fact that patients with mild leptospirosis maintained their IgG levels at acute serum sample, collected approximately 5 days after the onset of symptoms, and at convalescent sample, collected at least 13 days later, suggests that they mounted an anamnestic response due to a secondary leptospiral infection. In contrast, patients with the severe form showed an antibody response typical of a primary infection, with an increase in IgG levels from acute to convalescent phases.

Our results indicate that the presence of antibodies anti-leptospiral proteins may be protective against clinical severe leptospirosis and that patients with mild disease might have had previous leptospiral infection(s). However, numerous aspects can affect the host immune response against an infectious agent, including the inoculum size. Patients with severe clinical presentations might have been infected with a higher bacterial load than patients who presented the mild form, developing thereby a more intense immune response. In addition, we can't affirm that any of the patients enrolled in the present study had never been exposed to leptospira before since leptospirosis is highly endemic in their community. Nonetheless, there is a need of studies of this kind to help elucidate the immune response associated with naturally-acquired leptospirosis and we believe our work brings relevant information to the field.

## Supporting information

S1 Fig Representative agarose gels and microarray images. (A) Representative agarose gels of PCR amplifications and plasmid mini-preparations. All PCR amplicons and plasmid mini-preparations were verified in agarose gels before microarray production. (B) Two subarrays showing His (left) and HA (right) probing for protein expression evaluation. Each microarray chip contained 16 subarrays. Highlighted spots correspond to IVTT control reactions (NoDNA, red boxes), IgGmix (blue) and human IgM (green).
(TIF)

S2 Fig Patients cumulative IgM nd IgG reactivity against sero-reactive antigens. Summed average signal intensity is shown (y-axis) as the number of antigens (x-axis) increases. Cumulative reactivity is shown for patients with (left) and mild (right) illness against all 478 reactive antigens (up) and the 36 serodiagnostic antigens.
(TIF)

S3 Fig Heatmap of the antibody fold-change from acute to convalescent illness for 10 immunodominant antigens. Fold-change is represented according to the colorized scale with red strongest, black in-between and green weakest. Antigens are in rows; patient samples are in columns, grouped by clinical presentation and sorted from left to right by increasing average antigen intensity within each group. IgM fold-change is shown on the left; IgG fold-change is shown on the right. The five outliers in the mild group are highlighted with star (*).
(TIF)

S1 Table Proteins not represented on the microarrays. (DOCX)

S2 Table Acute phase serodiagnostic antigens identified in patients with mild and severe leptospirosis. (DOCX)

S3 Table Convalescent phase serodiagnostic antigens identified in patients with mild and severe leptospirosis. (DOCX)

S4 Table Clinical characteristics for mild leptospirosis patients with distinct antibody kinetics. (DOCX)

S1 Results (DOCX)
