# Supplementary figures and images for: Distinct antibody responses of patients with mild and severe leptospirosis determined by whole proteome microarray analysis
Source: PLoS Negl Trop Dis. 2017 Jan 31;11(1):e0005349. doi: 10.1371/journal.pntd.0005349 (PMC5302828; doi:10.1371/journal.pntd.0005349)

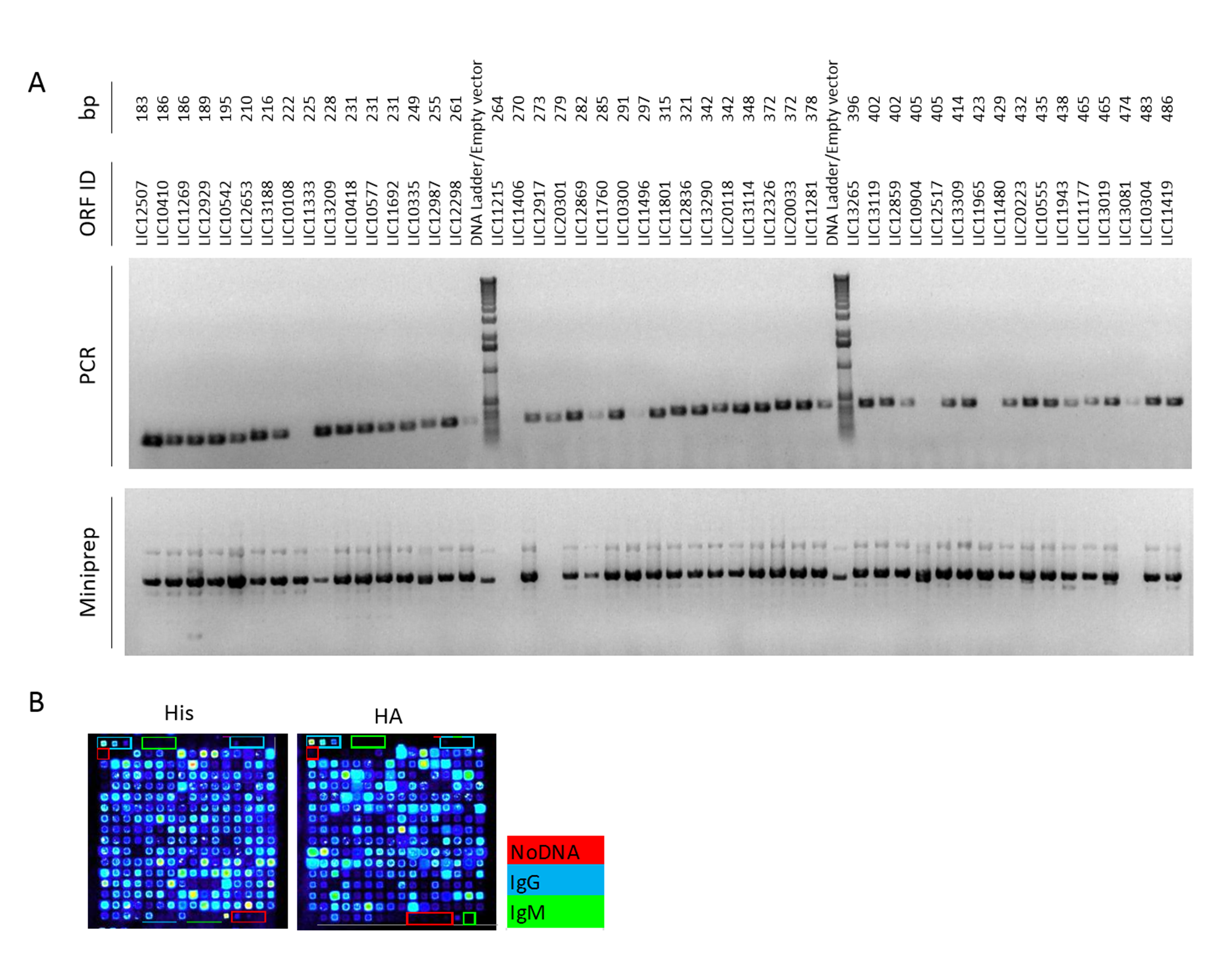

Supplement: S1 Fig — (A) Representative agarose gels of PCR amplifications and plasmid mini-preparations. All PCR amplicons and plasmid mini-preparations were verified in agarose gels before microarray production. (B) Two subarrays showing His (left) and HA (right) probing for protein expression evaluation. Each microarray chip contained 16 subarrays. Highlighted spots correspond to IVTT control reactions (NoDNA, red boxes), IgGmix (blue) and human IgM (green). (TIF) [file pntd.0005349.s001.tif]

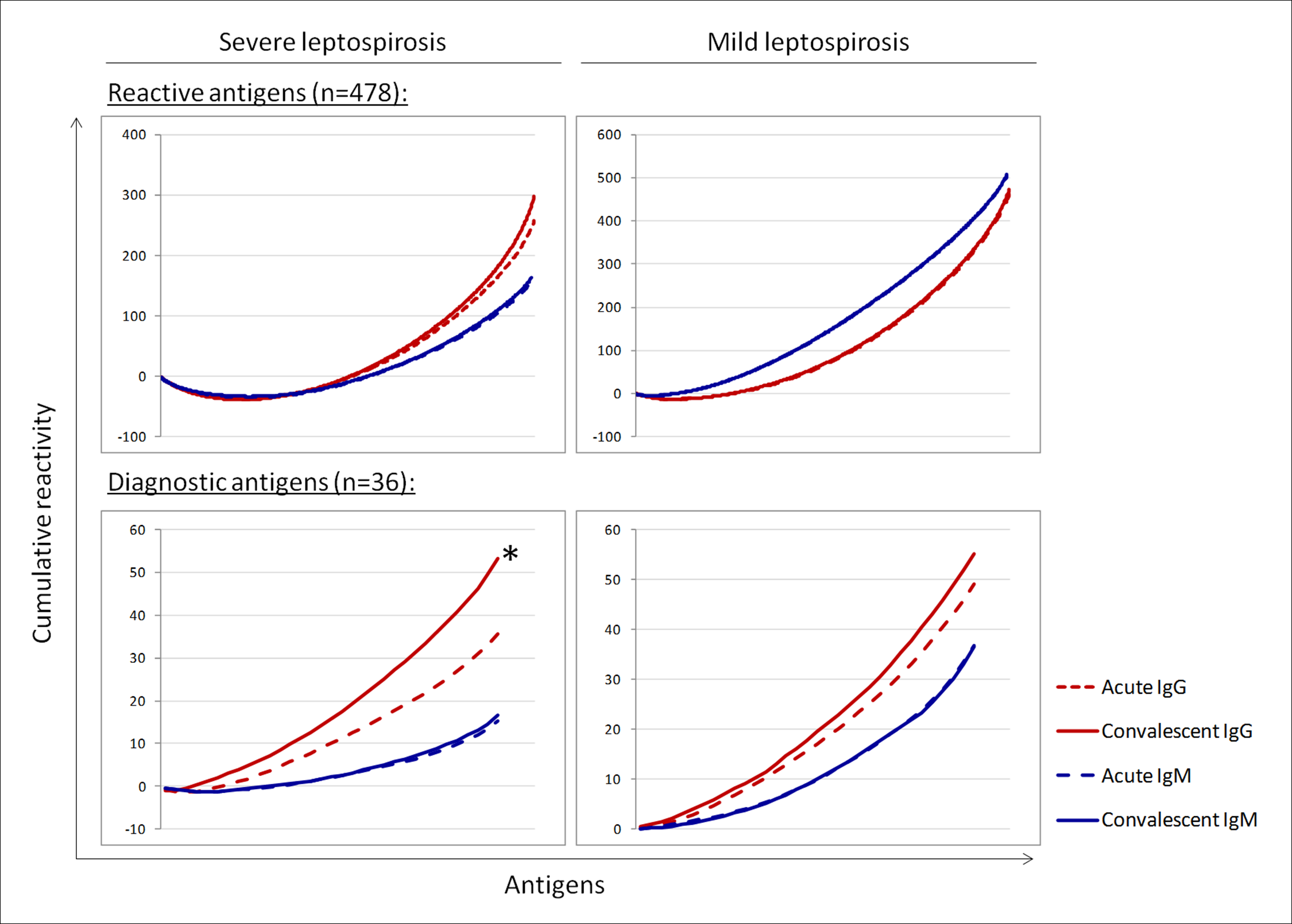

Supplement: S2 Fig — Summed average signal intensity is shown (y-axis) as the number of antigens (x-axis) increases. Cumulative reactivity is shown for patients with (left) and mild (right) illness against all 478 reactive antigens (up) and the 36 serodiagnostic antigens. (TIF) [file pntd.0005349.s002.tif]

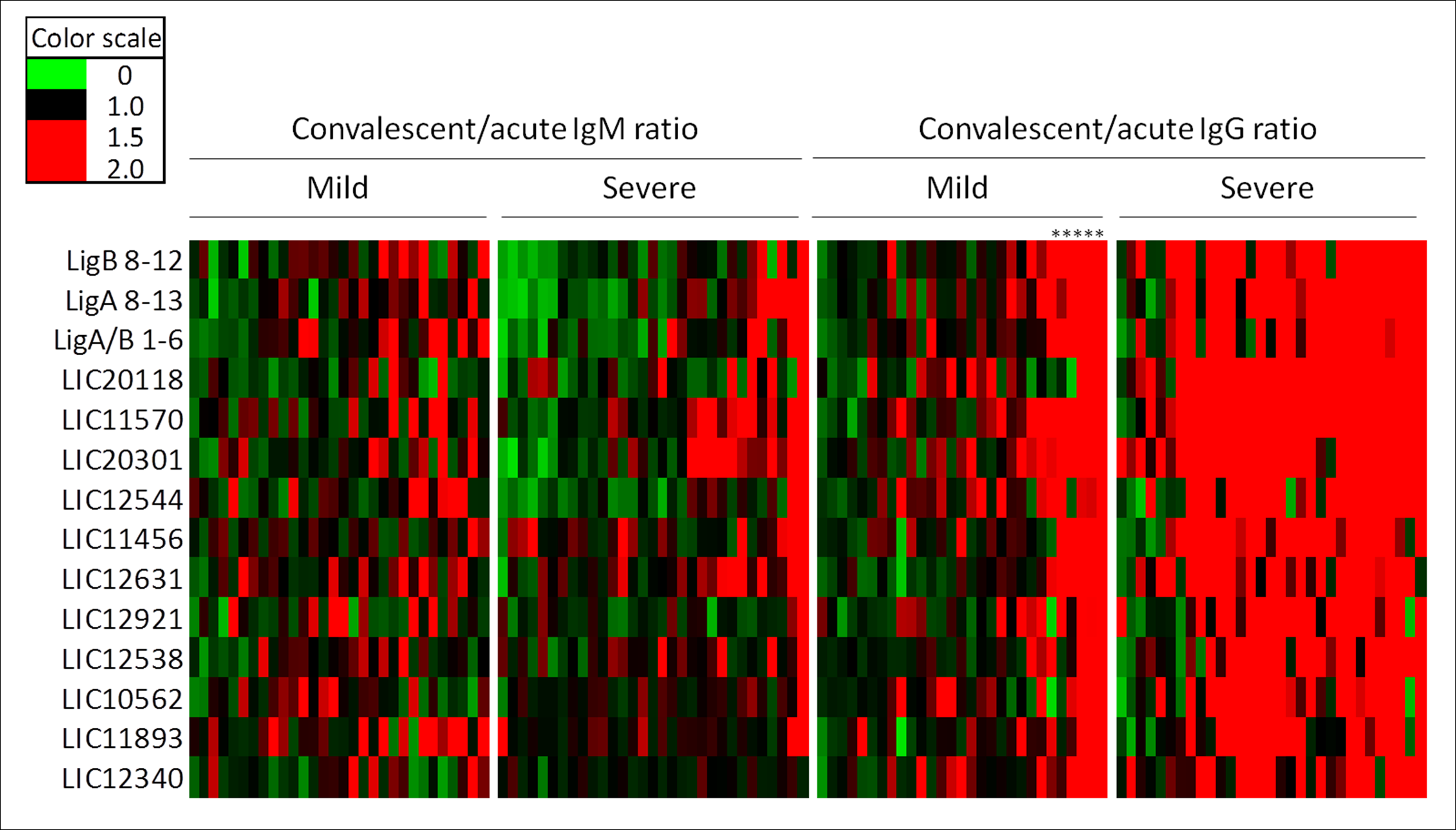

Supplement: S3 Fig — Fold-change is represented according to the colorized scale with red strongest, black in-between and green weakest. Antigens are in rows; patient samples are in columns, grouped by clinical presentation and sorted from left to right by increasing average antigen intensity within each group. IgM fold-change is shown on the left; IgG fold-change is shown on the right. The five outliers in the mild group are highlighted with star (*). (TIF) [file pntd.0005349.s003.tif]
