# Supplementary material for: Distinct antibody responses of patients with mild and severe leptospirosis determined by whole proteome microarray analysis
Source: PLoS Negl Trop Dis. 2017 Jan 31;11(1):e0005349. doi: 10.1371/journal.pntd.0005349 (PMC5302828; doi:10.1371/journal.pntd.0005349)
Supplement: S1 Table — (DOCX) [file pntd.0005349.s004.docx]

**S1 Table. Proteins not represented on the microarrays.**

| **Locustag** | **Accession** | **Product Name** |
| --- | --- | --- |
| LIC10889 | YP_000866.1 | TonB |
| LIC11008 | YP_000981.1 | hypothetical protein LIC11008 |
| LIC11109 | YP_001079.1 | hypothetical protein LIC11109 |
| LIC11352 | YP_001316.1 | LipL32 |
| LIC11364 | YP_001328.1 | short-chain dehydrogenase |
| LIC11885 | YP_001834.1 | putative lipoprotein |
| LIC12227 | YP_002162.1 | hypothetical protein LIC12227 |
| LIC12322 | YP_002256.1 | glutaconate CoA transferase-like protein |
| LIC13131 | YP_003039.1 | hypothetical protein LIC13131 |
| LIC20250 | YP_003634.1 | peptidoglycan-associated cytoplasmic membrane protein |
| LIC20254 | YP_003638.1 | response regulator |
| LIC10415 | YP_000401.1 | hypothetical protein LIC10415 |
| LIC20044 | YP_003436.1 | heat shock protein 90 |
| LIC13136 | YP_003044.1 | acyl-CoA dehydrogenase |
| LIC11791 | YP_001743.1 | ATP-dependent DNA helicase |
| LIC10772 | YP_000752.1 | hypothetical protein LIC10772 |
| LIC10037 | YP_000037.1 | GMC oxidoreductase |
| LIC11499 | YP_001460.1 | hypothetical protein LIC11499 |
| LIC11898 | YP_001847.1 | pyruvate dehydrogenase subunit beta |
| LIC10958 | YP_000932.1 | alcohol dehydrogenase |
| LIC11620 | YP_001578.1 | DNA repair protein |
| LIC11707 | YP_001659.1 | fructose-1,6-bisphosphatase |
| LIC10838 | YP_000815.1 | glyoxalase |
| LIC12414 | YP_002345.1 | hypothetical protein LIC12414 |
| LIC12958 | YP_002871.1 | hypothetical protein LIC12958 |
| LIC11423 | YP_001385.1 | glycine rich RNA-binding protein |
| LIC12744 | YP_002664.1 | NADH dehydrogenase subunit I D |
| LIC12251 | YP_002186.1 | L-aspartate oxidase |
| LIC13194 | YP_003102.1 | cholesterol oxidase |
| LIC11500 | YP_001461.1 | methionine aminopeptidase |
| LIC10712 | YP_000696.1 | hypothetical protein LIC10712 |
| LIC11851 | YP_001800.1 | cytoplasmic membrane protein |
| LIC12679 | YP_002601.1 | thiamine biosynthesis protein ThiC |
| LIC11854 | YP_001803.1 | putative methyltransferase |
| LIC11989 | YP_001928.1 | hypothetical protein LIC11989 |
| LIC10163 | YP_000155.1 | hypothetical protein LIC10163 |
| LIC10241 | YP_000232.1 | hypothetical protein LIC10241 |
| LIC11514 | YP_001473.1 | hypothetical protein LIC11514 |
| LIC12331 | YP_002263.1 | hypothetical protein LIC12331 |
| LIC12675 | YP_002597.1 | hypothetical protein LIC12675 |
| LIC13261 | YP_003168.1 | hypothetical protein LIC13261 |
| LIC20156 | YP_003547.1 | hypothetical protein LIC20156 |
| LIC11134 | YP_001104.2 | histone deacetylase |
| LIC10434 | YP_000419.1 | hypothetical protein LIC10434 |
| LIC10723 | YP_000707.1 | flagellar hook-associated protein FliD |
| LIC11062 | YP_001032.1 | hypothetical protein LIC11062 |
| LIC20192 | YP_003583.1 | hypothetical protein LIC20192 |
| LIC11433 | YP_001395.1 | histidine kinase sensor protein |
| LIC13032 | YP_002945.1 | hypothetical protein LIC13032 |
| LIC12646 | YP_002569.1 | membrane carboxypeptidase |
| LIC10298 | YP_000288.1 | flagellar basal body rod protein FlgC |
| LIC12993 | YP_002906.1 | hypothetical protein LIC12993 |
| LIC10021 | YP_000021.1 | putative lipoprotein |
| LIC10137 | YP_000133.1 | [protein-PII] uridylyltransferase |
| LIC10417 | YP_000403.1 | hypothetical protein LIC10417 |
| LIC10774 | YP_000754.1 | putative lipoprotein |
| LIC10784 | YP_000763.1 | transposase, IS1501 |
| LIC10785 | YP_000764.1 | transposase, IS1501 |
| LIC10865 | YP_000842.1 | hypothetical protein LIC10865 |
| LIC11033 | YP_001003.1 | hypothetical protein LIC11033 |
| LIC11225 | YP_001194.1 | hypothetical protein LIC11225 |
| LIC11242 | YP_001211.1 | F0F1 ATP synthase subunit gamma |
| LIC11305 | YP_001271.1 | hypothetical protein LIC11305 |
| LIC11306 | YP_001272.1 | hypothetical protein LIC11306 |
| LIC11321 | YP_001287.1 | hypothetical protein LIC11321 |
| LIC11382 | YP_001346.1 | hypothetical protein LIC11382 |
| LIC11432 | YP_001394.1 | histidine kinase response regulator hybrid protein |
| LIC11587 | YP_001546.1 | ribonucleotide-diphosphate reductase subunit alpha |
| LIC11690 | YP_001642.1 | hypothetical protein LIC11690 |
| LIC11760 | YP_001712.1 | 50S ribosomal protein L28 |
| LIC11995 | YP_001934.1 | hypothetical protein LIC11995 |
| LIC12111 | YP_002045.2 | 30S ribosomal protein S18 |
| LIC12159 | YP_002094.1 | hypothetical protein LIC12159 |
| LIC12249 | YP_002184.1 | hypothetical protein LIC12249 |
| LIC12385 | YP_002317.1 | hypothetical protein LIC12385 |
| LIC12525 | YP_002453.1 | putative lipoprotein |
| LIC12558 | YP_002485.1 | hypothetical protein LIC12558 |
| LIC12637 | YP_002560.1 | 50S ribosomal protein L31 |
| LIC12839 | YP_002755.1 | aspartate carbamoyltransferase catalytic subunit |
| LIC12855 | YP_002771.1 | 50S ribosomal protein L30 |
| LIC12860 | YP_002776.1 | 30S ribosomal protein S14 |
| LIC12865 | YP_002781.1 | 50S ribosomal protein L29 |
| LIC13013 | YP_002926.1 | hypothetical protein LIC13013 |
| LIC13034 | YP_002947.1 | hypothetical protein LIC13034 |
| LIC13109 | YP_003018.1 | hypothetical protein LIC13109 |
| LIC13163 | YP_003071.1 | 3-methyl-2-oxobutanoate hydroxymethyltransferase |
| LIC13182 | YP_003090.1 | glycerophosphoryl diester phosphodiesterase |
| LIC13221 | YP_003129.1 | Fe-S oxidoreductase |
| LIC13391 | YP_003294.1 | hypothetical protein LIC13391 |
| LIC13422 | YP_003325.1 | hydrogenase subunit |
| LIC14000 | YP_003858659.1 | hypothetical protein LIC14000 |
| LIC20065 | YP_003457.1 | acyl carrier protein |
| LIC20263 | YP_003647.1 | hypothetical protein LIC20263 |
| LIC20300 | YP_003858670.1 | hypothetical protein LIC20300 |
| LIC12693 | YP_002614.1 | hypothetical protein LIC12693 |
| LIC13301 | YP_003206.1 | acetyl-CoA acetyltransferase |
| LIC10583 | YP_000567.1 | acyl-CoA dehydrogenase |
| LIC13009 | YP_002922.1 | acyl-CoA dehydrogenase |
| LIC10445 | YP_000430.1 | glucosamine--fructose-6-phosphate aminotransferase |
| LIC11571 | YP_001530.1 | general secretory pathway protein E |
| LIC20175 | YP_003566.1 | dnaK suppressor |
| LIC12026 | YP_001964.1 | bifunctional 5,10-methylene-tetrahydrofolate dehydrogenase/ 5,10-methylene-tetrahydrofolate cyclohydrolase |
| LIC12535 | YP_002463.1 | riboflavin synthase subunit alpha |
| LIC11602 | YP_001560.1 | hypothetical protein LIC11602 |
| LIC13020 | YP_002933.2 | hypothetical protein LIC13020 |
| LIC10641 | YP_000625.1 | diguanylate phosphodiesterase |
| LIC20259 | YP_003643.1 | hypothetical protein LIC20259 |
| LIC10485 | YP_000469.1 | hypothetical protein LIC10485 |
| LIC13110 | YP_003019.1 | histidine kinase response regulator hybrid protein |
| LIC12700 | YP_002621.1 | metalloprotease |
| LIC12163 | YP_002098.1 | hypothetical protein LIC12163 |
| LIC12785 | YP_002702.1 | ribosomal RNA large subunit methyltransferase N |
| LIC12302 | YP_002236.1 | putative DNA-binding/iron metalloprotein/AP endonuclease |
| LIC13280 | YP_003187.1 | MaoC family protein |
| LIC10668 | YP_000652.1 | Mrr |
| LIC13457 | YP_003360.1 | hypothetical protein LIC13457 |
| LIC10561 | YP_000545.1 | hypothetical protein LIC10561 |
| LIC11773 | YP_001725.1 | N-(5'-phosphoribosyl)anthranilate isomerase |
| LIC10376 | YP_000362.1 | hypothetical protein LIC10376 |
| LIC10454 | YP_000438.1 | hypothetical protein LIC10454 |
| LIC13389 | YP_003292.1 | DNA mismatch repair protein |
| LIC12428 | YP_002359.1 | ABC transporter ATP-binding protein |
| LIC12692 | YP_002613.1 | hypothetical protein LIC12692 |
| LIC10139 | YP_000135.1 | hypothetical protein LIC10139 |
| LIC10154 | YP_000147.1 | 50S ribosomal protein L34 |
| LIC10309 | YP_000299.1 | glycine dehydrogenase |
| LIC10689 | YP_000673.1 | phosphate transport system protein |
| LIC10767 | YP_000747.1 | hypothetical protein LIC10767 |
| LIC10821 | YP_000798.1 | putative lipoprotein |
| LIC10882 | YP_000859.1 | hypothetical protein LIC10882 |
| LIC10943 | YP_000917.1 | hypothetical protein LIC10943 |
| LIC10998 | YP_000972.1 | hypothetical protein LIC10998 |
| LIC11172 | YP_001142.1 | hypothetical protein LIC11172 |
| LIC11215 | YP_001184.1 | hypothetical protein LIC11215 |
| LIC11243 | YP_001212.1 | F0F1 ATP synthase subunit beta |
| LIC11502 | YP_001463.1 | hypothetical protein LIC11502 |
| LIC11540 | YP_001499.1 | CTP synthetase |
| LIC11827 | YP_001778.1 | lipopolysaccharide biosynthesis glycosyltransferase |
| LIC11867 | YP_001816.1 | UDP-N-acetylmuramoylalanyl-D-glutamate--2,6-diaminopimelate ligase |
| LIC12234 | YP_002169.1 | hypothetical protein LIC12234 |
| LIC12462 | YP_002390.1 | 50S ribosomal protein L35 |
| LIC12472 | YP_002400.1 | acetyltransferase |
| LIC12634 | YP_002557.1 | hypothetical protein LIC12634 |
| LIC12711 | YP_002632.1 | N-acetylglutamate synthase/acetyltransferase |
| LIC12912 | YP_002828.1 | hypothetical protein LIC12912 |
| LIC12914 | YP_002830.1 | hypothetical protein LIC12914 |
| LIC12970 | YP_002883.1 | hypothetical protein LIC12970 |
| LIC13148 | YP_003056.1 | hypothetical protein LIC13148 |
| LIC13177 | YP_003085.1 | exonuclease |
| LIC13252 | YP_003159.1 | methylamine utilization ferredoxin-type protein |
| LIC13305 | YP_003210.1 | putative lipoprotein |
| LIC13349 | YP_003254.1 | hypothetical protein LIC13349 |
| LIC13427 | YP_003330.1 | response regulator |
| LIC20180 | YP_003571.1 | response regulator |
| LIC12357 | YP_002289.1 | elongation factor EF-G |
| LIC12616 | YP_002539.1 | hypothetical protein LIC12616 |
| LIC10396 | YP_000382.1 | acetyl-CoA acetyltransferase |
| LIC10522 | YP_000506.1 | oxidoreductase family protein |
| LIC20209 | YP_003598.1 | methylmalonyl-CoA mutase |
| LIC11555 | YP_001514.2 | 30S ribosomal protein S16 |
| LIC12551 | YP_002478.1 | acyl-CoA dehydrogenase |
| LIC12027 | YP_001965.1 | hypothetical protein LIC12027 |
| LIC10538 | YP_000522.1 | ABC transporter ATP-binding protein |
| LIC13219 | YP_003127.2 | phenylalanyl-tRNA synthetase subunit beta |
| LIC12792 | YP_002709.1 | pantothenate kinase |
| LIC11067 | YP_001037.1 | hypothetical protein LIC11067 |
| LIC10344 | YP_000334.1 | anti-sigma factor antagonist |
| LIC11741 | YP_001693.1 | hypothetical protein LIC11741 |
| LIC10222 | YP_000213.1 | DNA polymerase III subunit alpha |
| LIC11536 | YP_001495.1 | hypothetical protein LIC11536 |
| LIC13372 | YP_003277.1 | phosphoadenosine phosphosulphate reductase |
| LIC12173 | YP_002108.1 | N-acetylneuraminate synthase |
| LIC13464 | YP_003367.1 | carboxypeptidase I |
| LIC13021 | YP_002934.1 | hypothetical protein LIC13021 |
| LIC10662 | YP_000646.1 | hypothetical protein LIC10662 |
| LIC11811 | YP_001762.1 | hypothetical protein LIC11811 |
| LIC13331 | YP_003236.1 | histidine kinase sensor protein |
| LIC12268 | YP_002203.1 | putative glycolate oxidase |
| LIC13147 | YP_003055.1 | hypothetical protein LIC13147 |
| LIC12371 | YP_002303.1 | hypothetical protein LIC12371 |
| LIC11844 | YP_001793.1 | hypothetical protein LIC11844 |
| LIC10040 | YP_000040.1 | polysialic acid capsule expression protein |
| LIC10068 | YP_000068.1 | hypothetical protein LIC10068 |
| LIC12600 | YP_002523.1 | hypothetical protein LIC12600 |
| LIC10678 | YP_000662.1 | response regulator |
| LIC13077 | YP_002989.1 | hypothetical protein LIC13077 |
| LIC11311 | YP_001277.1 | acetyl-CoA C-acyltransferase |
| LIC11421 | YP_001383.1 | phosphate sodium symporter |
| LIC12014 | YP_001953.1 | hypothetical protein LIC12014 |
| LIC12298 | YP_002232.1 | hypothetical protein LIC12298 |
| LIC11879 | YP_001828.1 | hypothetical protein LIC11879 |
| LIC12178 | YP_002113.1 | hypothetical protein LIC12178 |
| LIC13302 | YP_003207.1 | hypothetical protein LIC13302 |
| LIC12097 | YP_002031.1 | histidine kinase sensor protein |
| LIC12539 | YP_002467.1 | hypothetical protein LIC12539 |
| LIC10323 | YP_000313.1 | 2,3-bisphosphoglycerate-independent phosphoglycerate mutase gene |
| LIC11045 | YP_001015.1 | adenylate or guanylate cyclase |
| LIC11390 | YP_001353.1 | hypothetical protein LIC11390 |
| LIC11624 | YP_001582.1 | ATP-dependent DNA helicase |
| LIC10051 | YP_000051.1 | 7,8-dihydropteroate synthase protein |
| LIC20222 | YP_003611.1 | hypothetical protein LIC20222 |
| LIC10950 | YP_000924.1 | sigma factor regulatory protein |
| LIC13218 | YP_003126.1 | carbonic anhydrase/acetyltransferase |
| LIC10161 | YP_000153.1 | hypothetical protein LIC10161 |
| LIC10227 | YP_000218.1 | hypothetical protein LIC10227 |
| LIC13118 | YP_003027.1 | amino-sugar biosynthesis protein |
| LIC10529 | YP_000513.1 | hypothetical protein LIC10529 |
| LIC11748 | YP_001700.1 | hypothetical protein LIC11748 |
| LIC12019 | YP_001957.1 | ATP-dependent helicase |
| LIC10200 | YP_000191.1 | glycosyltransferase |
| LIC10131 | YP_000127.1 | cell cycle protein |
| LIC10189 | YP_000180.1 | hypothetical protein LIC10189 |
| LIC10083 | YP_000082.1 | hypothetical protein LIC10083 |
| LIC10217 | YP_000208.1 | hypothetical protein LIC10217 |
| LIC10255 | YP_000246.1 | hypothetical protein LIC10255 |
| LIC10448 | YP_000432.1 | 30S ribosomal protein S20 |
| LIC10563 | YP_000547.1 | SenC |
| LIC10639 | YP_000623.1 | hypothetical protein LIC10639 |
| LIC10688 | YP_000672.1 | hypothetical protein LIC10688 |
| LIC10762 | YP_000742.1 | 30S ribosomal protein S9 |
| LIC10807 | YP_000784.1 | glutathione S-transferase |
| LIC10867 | YP_000844.1 | hypothetical protein LIC10867 |
| LIC10917 | YP_000892.1 | hypothetical protein LIC10917 |
| LIC10955 | YP_000929.1 | 1-hydroxy-2-methyl-2-(E)-butenyl 4-diphosphate synthase |
| LIC11002 | YP_000975.1 | IS1533 transposase |
| LIC11126 | YP_001096.1 | GGDEF family protein |
| LIC11178 | YP_001148.1 | hypothetical protein LIC11178 |
| LIC11226 | YP_001195.1 | hypothetical protein LIC11226 |
| LIC11269 | YP_001238.1 | hypothetical protein LIC11269 |
| LIC11308 | YP_001274.1 | acetylglutamate kinase |
| LIC11322 | YP_001288.1 | hypothetical protein LIC11322 |
| LIC11323 | YP_001289.1 | methyltransferase DNA modification enzyme |
| LIC11339 | YP_001303.1 | deoxyribodipyrimidine photolyase |
| LIC11497 | YP_001458.1 | methyltransferase |
| LIC11717 | YP_001669.1 | excinuclease ABC subunit A |
| LIC11746 | YP_001698.1 | N-acetyl-gamma-glutamyl-phosphate reductase |
| LIC11774 | YP_001726.1 | DNA mismatch repair protein MutS |
| LIC12335 | YP_002267.1 | serine hydroxymethyltransferase |
| LIC12370 | YP_002302.1 | hypothetical protein LIC12370 |
| LIC12386 | YP_002318.1 | drug/metabolite exporter |
| LIC12406 | YP_002338.1 | hypothetical protein LIC12406 |
| LIC12417 | YP_002348.1 | GTP-binding protein EngA |
| LIC12461 | YP_002389.1 | 50S ribosomal protein L20 |
| LIC12650 | YP_002573.1 | hypothetical protein LIC12650 |
| LIC12670 | YP_002592.1 | adenylate/guanylate cyclase |
| LIC12688 | YP_002609.1 | exodeoxyribonuclease v subunit gamma |
| LIC12697 | YP_002618.1 | hypothetical protein LIC12697 |
| LIC12750 | YP_002670.1 | NADH dehydrogenase I subunit L |
| LIC12752 | YP_002672.1 | NADH dehydrogenase I subunit N |
| LIC12753 | YP_002673.1 | hypothetical protein LIC12753 |
| LIC12784 | YP_002701.1 | putative lipoprotein |
| LIC12825 | YP_002741.1 | hypothetical protein LIC12825 |
| LIC12869 | YP_002785.1 | 30S ribosomal protein S19 |
| LIC13081 | YP_002993.1 | hypothetical protein LIC13081 |
| LIC13129 | YP_003037.1 | methionyl-tRNA synthetase |
| LIC13291 | YP_003198.1 | ankyrin domain-containing protein |
| LIC13304 | YP_003209.1 | hypothetical protein LIC13304 |
| LIC13317 | YP_003222.1 | hypothetical protein LIC13317 |
| LIC13320 | YP_003225.1 | thermolysin precursor |
| LIC13322 | YP_003227.1 | thermolysin precursor |
| LIC13430 | YP_003333.1 | hypothetical protein LIC13430 |
| LIC13468 | YP_003370.1 | hypothetical protein LIC13468 |
| LIC14005 | YP_003858660.1 | hypothetical protein LIC14005 |
| LIC20255 | YP_003639.1 | hypothetical protein LIC20255 |
| LIC10382 | YP_000368.1 | acyl-CoA dehydrogenase |
| LIC10543 | YP_000527.1 | acyl-CoA dehydrogenase |
| LIC11173 | YP_001143.1 | hypothetical protein LIC11173 |
| LIC12505 | YP_002433.1 | response regulator |
| LIC12762 | YP_002682.1 | hypothetical protein LIC12762 |
| LIC20198 | YP_003587.1 | hypothetical protein LIC20198 |
| LIC11257 | YP_001226.1 | exopolyphosphatase |
| LIC12350 | YP_002282.1 | glycyl-tRNA synthetase |
| LIC13448 | YP_003351.1 | carbon storage regulator |
| LIC10174 | YP_000166.1 | hypothetical protein LIC10174 |
| LIC10896 | YP_000873.1 | TonB-dependent outer membrane receptor |
| LIC20009 | YP_003401.1 | porphobilinogen deaminase |
| LIC12701 | YP_002622.1 | polynucleotide phosphorylase/polyadenylase |
| LIC12421 | YP_002352.1 | hypothetical protein LIC12421 |
| LIC12205 | YP_002140.1 | GDP-l-fucose synthetase |
| LIC11483 | YP_001444.1 | hypothetical protein LIC11483 |
| LIC20088 | YP_003480.1 | diphosphate--fructose-6-phosphate 1-phosphotransferase |
| LIC10151 | YP_000145.1 | hypothetical protein LIC10151 |
| LIC12536 | YP_002464.1 | hypothetical protein LIC12536 |
| LIC10350 | YP_000339.1 | hypothetical protein LIC10350 |
| LIC12413 | YP_002344.1 | hypothetical protein LIC12413 |
| LIC20265 | YP_003649.1 | hypothetical protein LIC20265 |
| LIC13308 | YP_003213.1 | hypothetical protein LIC13308 |
| LIC10322 | YP_000312.1 | hypothetical protein LIC10322 |
| LIC10457 | YP_000441.1 | hypothetical protein LIC10457 |
| LIC10556 | YP_000540.1 | hypothetical protein LIC10556 |
| LIC11034 | YP_001004.1 | hypothetical protein LIC11034 |
| LIC11246 | YP_001215.1 | hypothetical protein LIC11246 |
| LIC11248 | YP_001217.1 | alginate o-acetyltransferase |
| LIC11453 | YP_001414.1 | histidinol dehydrogenase |
| LIC11460 | YP_001421.1 | aspartyl/glutamyl-tRNA amidotransferase subunit C |
| LIC11558 | YP_001517.1 | tRNA (guanine-N(1)-)-methyltransferase/unknown domain fusion protein |
| LIC11647 | YP_001605.1 | pseudouridylate synthase |
| LIC11655 | YP_001611.1 | bifunctional phosphoribosylaminoimidazolecarboxamide formyltransferase/IMP cyclohydrolase |
| LIC11869 | YP_001818.1 | S-adenosyl-methyltransferase |
| LIC12025 | YP_001963.1 | asparaginyl-tRNA synthetase |
| LIC12562 | YP_002489.1 | putative ring hydroxylating dioxygenase alpha-subunit |
| LIC13404 | YP_003307.1 | acetyl-CoA synthetase |
| LIC20055 | YP_003447.1 | hypothetical protein LIC20055 |
| LIC20110 | YP_003502.1 | hypothetical protein LIC20110 |
| LIC10464 | YP_000448.1 | Ig-like repeat-containing protein |
| LIC10465 | YP_000449.1 | Ig-like repeat-containing protein |
| LIC11739 | YP_001691.1 | hypothetical protein LIC11739 |
| LIC11026 | YP_000996.1 | hypothetical protein LIC11026 |
| LIC12602 | YP_002525.1 | hypothetical protein LIC12602 |
| LIC11755 | YP_001707.1 | hypothetical protein LIC11755 |
| LIC12048 | YP_001983.1 | hypothetical protein LIC12048 |
