# Supplementary material for: Distinct antibody responses of patients with mild and severe leptospirosis determined by whole proteome microarray analysis
Source: PLoS Negl Trop Dis. 2017 Jan 31;11(1):e0005349. doi: 10.1371/journal.pntd.0005349 (PMC5302828; doi:10.1371/journal.pntd.0005349)
Supplement: S2 Table — (DOCX) [file pntd.0005349.s005.docx]

**S2 Table Acute phase serodiagnostic antigens identified in patients with mild and severe leptospirosis.**

| **Ag ID** | **Patients** | **Endemic controls** | **BHp-value** | **AUC** | **US naives** | **BHp-value** | **AUC** |
| --- | --- | --- | --- | --- | --- | --- | --- |
| *Mild patients* |  |  |  |  |  |  |  |
| LIC10973 | 1.640 (1.065/2.214) | -0.952 (-1.203/-0.701) | 4.24E-03 | 0.765 | -1.014 (-1.235/-0.793) | 9.89E-05 | 0.872 |
| LigB 8-12 | 3.757 (2.910/4.603) | 0.084 (-0.014/0.182) | 1.45E-04 | 0.809 | 0.287 (0.123/0.451) | 8.52E-05 | 0.847 |
| LigA/B 1-6^a^ | 3.899 (3.181/4.617) | 0.537 (0.424/0.651) | 1.70E-06 | 0.916 | 0.708 (0.272/1.144) | 1.01E-05 | 0.893 |
| LigA 8-13^a^ | 4.590 (3.993/5.186) | 1.688 (1.192/2.183) | 7.72E-05 | 0.851 | 1.413 (1.014/1.811) | 1.17E-05 | 0.873 |
| LIC11274 | 1.256 (0.864/1.649) | 0.395 (0.271/0.518) | 3.43E-02 | 0.784 | 0.358 (0.059/0.656) | 2.75E-02 | 0.798 |
| *Severe patients* | |  |  |  |  |  |  |
| LIC12544 | 1.438 (0.910/1.965) | -0.075 (-0.452/0.302) | 1.57E-03 | 0.844 | 0.008 (-0.414/0.430) | 4.13E-03 | 0.804 |
| LIC10486 | 1.925 (1.491/2.358) | 0.605 (0.317/0.893) | 9.36E-04 | 0.851 | 0.691 (0.224/1.158) | 9.37E-03 | 0.797 |
| LIC10024 | 1.005 (0.792/1.218) | 0.441 (0.277/0.606) | 5.29E-03 | 0.789 | 0.546 (0.458/0.634) | 6.04E-03 | 0.730 |
| LigA/B 1-6 | 3.033 (2.435/3.630) | 0.537 (0.424/0.651) | 1.63E-06 | 0.935 | 0.708 (0.272/1.144) | 3.25E-05 | 0.911 |
| LigA 8-13 | 3.444 (2.864/4.023) | 1.688 (1.192/2.183) | 1.94E-03 | 0.811 | 1.413 (1.014/1.811) | 1.03E-04 | 0.866 |
| LIC11591 | 1.145 (0.992/1.297) | 0.724 (0.619/0.830) | 1.94E-03 | 0.839 | 0.819 (0.702/0.936) | 2.38E-02 | 0.764 |
| LIC12731 | 1.425 (1.180/1.670) | 0.739 (0.473/1.004) | 1.24E-02 | 0.831 | 0.691 (0.617/0.764) | 1.24E-04 | 0.883 |
| LIC20077 | 1.088 (0.731/1.445) | 0.356 (0.048/0.663) | 3.65E-02 | 0.772 | 0.362 (0.090/0.634) | 3.19E-02 | 0.777 |
| LIC20276 | 1.474 (1.155/1.793) | 0.554 (0.414/0.694) | 6.94E-04 | 0.901 | 0.777 (0.647/0.907) | 5.58E-03 | 0.801 |

^a^Antigens detected in both mild and severe leptospirosis patients.
