# Supplementary material for: Distinct antibody responses of patients with mild and severe leptospirosis determined by whole proteome microarray analysis
Source: PLoS Negl Trop Dis. 2017 Jan 31;11(1):e0005349. doi: 10.1371/journal.pntd.0005349 (PMC5302828; doi:10.1371/journal.pntd.0005349)
Supplement: S3 Table — (DOCX) [file pntd.0005349.s006.docx]

**S3 Table. Convalescent phase serodiagnostic antigens identified in patients with mild and severe leptospirosis.**

| **Ag ID** | **Patients** | **Endemic controls** | **BHp-value** | **AUC** | **US naives** | **BHp-value** | **AUC** |
| --- | --- | --- | --- | --- | --- | --- | --- |
| *Mild patients* |  |  |  |  |  |  |  |
| LIC10462 | 1.614 (1.195/2.034) | -0.428 (-0.743/-0.113) | 1.80E-05 | 0.918 | -0.331 (-0.535/-0.126) | 2.15E-04 | 0.869 |
| LigA/B 1-6 ^a^ | 4.410 (3.741/5.079) | 0.537 (0.424/0.651) | 5.74E-09 | 0.944 | 0.708 (0.272/1.144) | 4.86E-08 | 0.929 |
| LigB 8-12 ^a^ | 4.454 (3.779/5.130) | 0.084 (-0.014/0.182) | 1.83E-08 | 0.955 | 0.287 (0.123/0.451) | 1.88E-08 | 0.946 |
| LigA 8-13 ^a^ | 4.973 (4.359/5.587) | 1.688 (1.192/2.183) | 3.11E-06 | 0.893 | 1.413 (1.014/1.811) | 4.73E-07 | 0.902 |
| LIC10713 | 1.052 (0.628/1.477) | -0.199 (-0.360/-0.038) | 6.31E-04 | 0.865 | -0.263 (-0.386/-0.140) | 8.40E-04 | 0.849 |
| LIC10973 | 1.991 (1.376/2.607) | -0.952 (-1.203/-0.701) | 3.67E-04 | 0.795 | -1.014 (-1.235/-0.793) | 1.19E-05 | 0.896 |
| LIC11570 ^a^ | 3.291 (2.686/3.897) | 0.565 (0.349/0.781) | 1.69E-05 | 0.855 | 0.705 (0.387/1.023) | 5.21E-04 | 0.822 |
| LIC11694 | 2.725 (2.205/3.244) | 0.596 (0.205/0.987) | 5.95E-03 | 0.808 | 0.350 (0.095/0.604) | 2.97E-03 | 0.792 |
| LIC13277 | 1.200 (0.726/1.674) | -0.523 (-0.696/-0.350) | 2.26E-03 | 0.809 | -1.166 (-1.407/-0.924) | 1.45E-06 | 0.909 |
| LIC1SPN3200s2 ^a^ | 2.352 (1.512/3.191) | 0.240 (0.112/0.367) | 1.50E-03 | 0.837 | 0.438 (0.227/0.649) | 1.52E-02 | 0.779 |
| *Severe patients* |  |  |  |  |  |  |  |
| LIC10215 | 1.030 (0.694/1.365) | -0.663 (-0.924/-0.403) | 4.17E-08 | 0.960 | -0.712 (-0.948/-0.476) | 8.30E-09 | 0.960 |
| LigA/B 1-6 ^a^ | 4.282 (3.897/4.666) | 0.537 (0.424/0.651) | 9.30E-17 | 1.000 | 0.708 (0.272/1.144) | 3.16E-11 | 0.990 |
| LigB 8-12 ^a^ | 2.435 (1.955/2.914) | 0.084 (-0.014/0.182) | 4.08E-09 | 0.988 | 0.287 (0.123/0.451) | 2.43E-08 | 0.978 |
| LigA 8-13 ^a^ | 5.210 (4.802/5.618) | 1.688 (1.192/2.183) | 7.94E-10 | 0.983 | 1.413 (1.014/1.811) | 5.10E-13 | 0.988 |
| LIC10486 | 2.761 (2.264/3.258) | 0.605 (0.317/0.893) | 1.29E-07 | 0.893 | 0.691 (0.224/1.158) | 2.23E-05 | 0.883 |
| LIC10562 | 2.098 (1.673/2.524) | 0.540 (0.279/0.801) | 6.81E-06 | 0.898 | 0.880 (0.337/1.422) | 1.53E-02 | 0.816 |
| LIC11222 | 2.164 (1.583/2.745) | 0.965 (0.508/1.423) | 2.39E-02 | 0.715 | 0.102 (-0.270/0.474) | 1.90E-05 | 0.871 |
| LIC11335 | 2.414 (1.790/3.037) | 0.594 (0.486/0.702) | 6.10E-05 | 0.844 | 0.887 (0.685/1.088) | 9.70E-04 | 0.784 |
| LIC11389 | 2.059 (1.700/2.417) | 0.893 (0.552/1.234) | 7.20E-04 | 0.836 | 1.173 (0.703/1.643) | 4.70E-02 | 0.747 |
| LIC11456 | 1.572 (1.214/1.929) | 0.344 (0.162/0.525) | 1.06E-05 | 0.898 | 0.373 (0.088/0.658) | 1.67E-04 | 0.871 |
| LIC11570^a^ | 3.319 (2.896/3.743) | 0.565 (0.349/0.781) | 2.70E-12 | 0.993 | 0.705 (0.387/1.023) | 2.65E-10 | 0.980 |
| LIC11653 | 1.057 (0.710/1.405) | 0.190 (0.040/0.339) | 8.76E-04 | 0.824 | 0.286 (0.056/0.517) | 7.55E-03 | 0.767 |
| LIC11893 | 1.645 (1.202/2.089) | 0.397 (0.165/0.629) | 2.64E-04 | 0.834 | 0.532 (0.301/0.763) | 1.27E-03 | 0.779 |
| LIC11966 | 2.582 (2.029/3.134) | 1.204 (0.923/1.485) | 1.20E-03 | 0.804 | 1.043 (0.753/1.332) | 3.66E-04 | 0.846 |
| LIC12180 | 1.113 (0.687/1.539) | 0.233 (-0.125/0.591) | 2.68E-02 | 0.730 | -0.067 (-0.435/0.301) | 2.54E-03 | 0.821 |
| LIC12340 | 1.956 (1.576/2.336) | 1.089 (0.768/1.409) | 1.49E-02 | 0.749 | 1.180 (0.964/1.397) | 1.08E-02 | 0.705 |
| LIC12500 | 1.423 (1.201/1.645) | 0.564 (0.486/0.642) | 4.90E-07 | 0.943 | 0.759 (0.626/0.893) | 2.07E-04 | 0.834 |
| LIC12538 | 1.939 (1.605/2.274) | 1.126 (0.820/1.432) | 1.23E-02 | 0.752 | 0.981 (0.768/1.194) | 4.64E-04 | 0.806 |
| LIC12544 | 2.690 (2.168/3.212) | -0.075 (-0.452/0.302) | 7.36E-09 | 0.958 | 0.008 (-0.414/0.430) | 4.95E-08 | 0.955 |
| LIC12631 | 3.609 (3.115/4.103) | 1.813 (1.364/2.263) | 1.09E-04 | 0.851 | 2.199 (1.734/2.663) | 2.89E-03 | 0.797 |
| LIC12731 | 1.442 (1.184/1.700) | 0.739 (0.473/1.004) | 7.95E-03 | 0.824 | 0.691 (0.617/0.764) | 8.95E-05 | 0.896 |
| LIC12921 | 1.982 (1.609/2.354) | 0.379 (0.248/0.511) | 5.26E-08 | 0.945 | 0.816 (0.579/1.052) | 1.35E-04 | 0.839 |
| LIC13314 | 1.782 (1.480/2.085) | 0.909 (0.667/1.151) | 1.04E-03 | 0.839 | 0.993 (0.819/1.166) | 1.03E-03 | 0.784 |
| LIC1SPN3200s2^a^ | 1.691 (1.218/2.164) | 0.240 (0.112/0.367) | 3.14E-05 | 0.938 | 0.438 (0.227/0.649) | 4.88E-04 | 0.876 |
| LIC20077 | 1.118 (0.757/1.480) | 0.356 (0.048/0.663) | 2.50E-02 | 0.777 | 0.362 (0.090/0.634) | 1.80E-02 | 0.779 |
| LIC20118 | 1.058 (0.709/1.408) | -1.233 (-1.516/-0.951) | 1.28E-10 | 0.970 | -1.438 (-1.651/-1.224) | 5.10E-13 | 0.988 |
| LIC20276 | 1.402 (1.078/1.727) | 0.554 (0.414/0.694) | 5.09E-04 | 0.888 | 0.777 (0.647/0.907) | 1.08E-02 | 0.792 |
| LIC20301 | 2.295 (1.854/2.737) | 0.283 (0.149/0.418) | 1.60E-08 | 0.948 | 0.156 (0.030/0.281) | 4.29E-09 | 0.955 |

^a^Antigens detected in both mild and severe leptospirosis patients.
