# Supplementary material for: Distinct antibody responses of patients with mild and severe leptospirosis determined by whole proteome microarray analysis
Source: PLoS Negl Trop Dis. 2017 Jan 31;11(1):e0005349. doi: 10.1371/journal.pntd.0005349 (PMC5302828; doi:10.1371/journal.pntd.0005349)
Supplement: S4 Table — (DOCX) [file pntd.0005349.s007.docx]

**S4 Table: Clinical characteristics for mild leptospirosis patients with distinct antibody kinetics.**

|  | **Median (IQR) or No (%)** | |  |  |
| --- | --- | --- | --- | --- |
|  | | **Mild patients** | **Mild outliers** | **p-value** |
| Number | | 25 | 5 |  |
| **Demographics** | |  |  |  |
| Sex | | 11.0 (44.0) | 5 (100) | 0.128 |
| Age | | 32.0 (18.0-44.0) | 11.0 (9.0-25.5) | 0.062 |
| **Clinical presentation** | |  |  |  |
| Days of symptoms | |  |  |  |
| Acute sample collection | | 6.0 (3.0-8.0) | 5.0 (5.0-10.5) | 0.633 |
| Convalescent sample collection | | 44.0 (22.0-86.5) | 41.0 (27.0-71.5) | 1.000 |
| **Laboratory confirmation** | |  |  |  |
| Acute MAT titer | | 0 (0-300) | 0 (0-800) | 0.628 |
| Convalescent MAT titer | | 200 (0-600) | 3200 (400-12800) | **0.031** |
