# Supplementary material for: Distinct antibody responses of patients with mild and severe leptospirosis determined by whole proteome microarray analysis
Source: PLoS Negl Trop Dis. 2017 Jan 31;11(1):e0005349. doi: 10.1371/journal.pntd.0005349 (PMC5302828; doi:10.1371/journal.pntd.0005349)
Supplement: S1 Results — (DOCX) [file pntd.0005349.s008.docx]

**Supplementary Information**

**Results**

**Protein Microarray Fabrication**

The complete *Leptospira interrogans* serovar Copenhageni ORFeome was submitted to PCR amplification and cloning using the high-throughput cloning strategy developed by our group [34]. Cloning efficiency was ∼94%, and all cloned ORFs were submitted to expression under a T7 promoter in the *E. coli* IVTT system. PCR and miniprep representative gels are shown in S1A Figure. Microarray probing with anti-His and anti-HA antibodies revealed an expression level of ∼91% of all proteins and fragments printed on the array (S1B Fig). A list of the proteins not represented on the microarrays is provided in S1 Table.
